# Visual working memory continues to develop through adolescence

**DOI:** 10.3389/fpsyg.2015.00696

**Published:** 2015-05-27

**Authors:** Elif Isbell, Keisuke Fukuda, Helen J. Neville, Edward K. Vogel

**Affiliations:** ^1^Department of Psychology, University of Oregon, Eugene, OR, USA; ^2^Department of Psychology, Vanderbilt University, Nashville, TN, USA

**Keywords:** visual working memory, working memory capacity, adolescence, cross-sectional, prolonged development

## Abstract

The capacity of visual working memory (VWM) refers to the amount of visual information that can be maintained in mind at once, readily accessible for ongoing tasks. In healthy young adults, the capacity limit of VWM corresponds to about three simple objects. While some researchers argued that VWM capacity becomes adult-like in early years of life, others claimed that the capacity of VWM continues to develop beyond middle childhood. Here we assessed whether VWM capacity reaches adult levels in adolescence. Using an adaptation of the visual change detection task, we measured VWM capacity estimates in 13-year-olds, 16-year-olds, and young adults. We tested whether the capacity estimates observed in early or later years of adolescence were comparable to the estimates obtained from adults. Our results demonstrated that the capacity of VWM continues to develop throughout adolescence, not reaching adult levels even in 16-year-olds. These findings suggest that VWM capacity displays a prolonged development, similar to the protracted trajectories observed in various other aspects of cognition.

## Introduction

The capacity of visual working memory (VWM) refers to the amount of visual information that can be maintained in the mind at once, readily available for rapid access (Luck and Vogel, 2013). It has been demonstrated that the capacity of VWM is highly limited (Luck and Vogel, 1997; Vogel and Machizawa, 2004; Xu and Chun, 2006; Awh et al., 2007; Zhang and Luck, 2008). When processing strategies are prevented or controlled in young adults, the capacity limit of VWM corresponds to about three simple objects (Pashler, 1988; Luck and Vogel, 1997; Cowan, 2001). Electrophysiological and functional magnetic resonance imaging studies provide further evidence for such limited capacity in VWM (Todd and Marois, 2004; Vogel and Machizawa, 2004). The estimates of such discrete capacity limits differ markedly across individuals (Vogel and Machizawa, 2004; Rouder et al., 2008). These capacity estimates strongly predict fluid intelligence in adults (Cowan et al., 2005, 2006; Fukuda et al., 2010; Unsworth et al., 2014). Furthermore, the VWM capacity yields high correlations with both intelligence and aptitude measures in children (Cowan et al., 2005, 2006). Understanding the development of this cognitive asset can shed light on both how such capacity limits emerge and how individual differences in crucial aspects of cognition unfold.

While superior performance in VWM tasks has been associated with favorable cognitive and educational outcomes, deficits in VWM have been observed in learning disabilities in reading (Reiter et al., 2005; Gathercole et al., 2006; Wang and Gathercole, 2013) and mathematics (McLean and Hitch, 1999; Ashkenazi et al., 2013; Szucs et al., 2013). In addition, VWM deficits have been documented in a wide spectrum of disorders, such as attention deficit and hyperactivity disorders (Martinussen et al., 2005; Lenartowicz et al., 2014) and schizophrenia (Goldman-Rakic, 1994; Silver et al., 2003; Lee and Park, 2005). Characterizing the typical developmental trajectory of VWM capacity limits can guide training and intervention efforts that target atypical populations in which VWM deficits are common. Profiling when and how VWM capacity matures can inform us about when the plasticity of VWM is less likely to be constrained due to maturation and the sensitive periods during which training and intervention efforts are more likely to be effective.

Several studies focused on investigating the typical developmental trajectory of VWM capacity. One of the common paradigms used in these developmental studies is the visual change detection task (Luck and Vogel, 1997). In this task, participants are briefly presented with a sample array of objects on each trial. Following a short retention period, a test array is presented and participants are asked to judge whether the sample array and the test array are identical or differ in one single item. The performance on these change-detection judgments is then used to determine the number of items that can be held in VWM, or in other words, an individual’s VWM capacity.

Variants of this paradigm were employed in infant studies to investigate the development of VWM during the first year of life (Ross-Sheehy et al., 2003, 2011; Oakes et al., 2006). For instance, Ross-Sheehy et al. (2003) used a looking preference paradigm to explore VWM capacity in infants. Infants were presented with two simultaneous displays of items, one with the same items streaming, and the other with one random item changing at each display. Looking preferences of infants were measured with the assumption that infants would show preferences for the changing displays as long as the number of items on the displays was within or near the capacity of their VWM. Four-and 6.5-month-old infants were reported to detect changes only at displays with one item, while infants as young as 10 months of age were found to prefer looking at changing displays that contained up to four items, but not six items. Based on this finding, it was concluded that infants reached almost an adult-like VWM capacity by the end of the first year. Employing similar tasks, Oakes et al. (2006) reported that even 7.5-month-olds were able to detect changes of color-location combinations in arrays of three objects. Together these results imply a rapid development in storing multiple objects in VWM during the first year of life.

However, contrary to the assertions that VWM capacity develops rapidly to the extent that it reaches almost adult levels in infancy, several studies argued a more protracted development, continuing at least through childhood. For instance, in a study with an adaptation of the change detection task for young children, 3- and 4-year-old children had lower VWM capacity estimates compared to 5- and 7-year-old children, and 5-year-olds performed significantly worse than 7-year-olds (Simmering, 2012). In a similar line of work, 5-year-olds were found to display lower capacity estimates than 10-year-olds across various set sizes (Riggs et al., 2006). These results suggest that VWM capacity continues to expand at least during early childhood and contradict the claims that VWM capacity becomes adult-like in infancy.

Furthermore, while Riggs et al. (2006) argued that VWM capacity reached adult levels of three to four items at 10 years of age, other studies reported lower capacity estimates for 10-year-olds compared to adults (Cowan et al., 2006; Riggs et al., 2011). Similarly, in a study comparing 10- to 12-year-old children to younger and older adults, children displayed higher capacity estimates than older adults only when the encoding times were short, but consistently showed lower capacity estimates than young adults (Sander et al., 2011). Moreover, in a cross-sectional study, Cowan et al. (2005) found lower VWM capacity in sixth-grade children compared to adults. In line with these findings, 12- to 16-year-olds adolescents were shown to have lower capacity estimates than adults in a change detection task when three target items were present (Spronk et al., 2012). Likewise, in a study that assessed VWM performance in a large sample of individuals between the ages of 8 and 75, a peak in VWM performance was reported around age 20 (Brockmole and Logie, 2013). Such converging evidence from independent studies suggests that VWM capacity is not adult-like in childhood and implies ongoing development at least during the early years of adolescence.

In the present study, we investigated whether VWM capacity shows a protracted development, extending from adolescence into adulthood. Adolescence is a time period during which the brain exhibits tremendous structural changes (Raznahan et al., 2011). The cortical regions that are involved in working memory processes such as the parietal cortex and prefrontal cortex (Curtis and D’Esposito, 2003; Todd and Marois, 2004) display maturational changes across adolescence (Giedd et al., 1999; Lebel and Beaulieu, 2011). Especially the prefrontal cortex displays changes in various features, such as the cortical thickness (Sowell et al., 2004; Lenroot and Giedd, 2006), gray matter density (Sowell et al., 2001), and white matter anisotropy (Nagy et al., 2004; Barnea-Goraly et al., 2005; Mabbott et al., 2006) well into adulthood. Based on these findings of prolonged brain development in adolescence, we expected to observe immature profiles of VWM in adolescents as compared to adults. Using an adaptation of the visual change detection task (Luck and Vogel, 1997), with set sizes 2, 4, and 6, we tested whether the capacity estimates obtained in early or later years of adolescence were comparable to the estimates attained from adults.

## Experiment 1

### Introduction

To investigate whether VWM capacity reaches adult levels in early or later years of adolescence, we recruited 13- and 16-year-old participants. Cowan et al. (2005) demonstrated that sixth grade children, ranging from 11 to 13 years of age, did not have capacity estimates as high as adults. Similarly, compared to adults, 12- to 16-year-old adolescents had lower capacity estimates when presented with three items (Spronk et al., 2012). Expecting to replicate these findings, we anticipated obtaining lower capacity estimates from 13-year-olds compared to adults. Moreover, based on the ongoing brain development throughout adolescence in regions associated with WM, we expected to observe lower capacity estimates also in 16-year-olds compared to adults.

### Method

#### Participants

Adolescent participants were recruited via the developmental database of the University of Oregon. All adolescent participants were middle school and high school students, attending a variety of schools in Eugene, Oregon. Parents were interviewed over the phone to ensure their children had normal or corrected-to-normal vision and were typically developing individuals with no neurological disorders, developmental delays, ADD/ADHD, learning disabilities, visual tracking problems, color blindness, depression or anxiety, and had never used any psychotropic drugs.

Adult participants were recruited via flyers from the University of Oregon. All adult participants were students at the university. Prior to participation, they were interviewed to ensure that they met all the criteria that were used to recruit adolescent participants.

The sample included three age groups: twenty-two 13-year-olds (*M* = 13.49 years; SD = 0.30; 13 females), twenty-two 16-year-olds (*M* = 16.58 years, SD = 0.34; 10 females), and 23 adults (*M* = 20.89 years, SD = 1.32; 14 females). One 13-year-old participant was not included in this final sample for performing below chance at higher set sizes, suggesting a high likelihood that this participant was not fully engaged in the task.

Maternal education levels were compared as a proxy for socio-economic status (SES). The mean maternal education level corresponded to “completed some college classes” across the age groups, which is the equivalent of some education beyond 12th grade and attendance to any post-secondary institution in the United States. No differences in maternal education were observed between groups [*F*(2,63) = 0.85, *p* = 0.43].

The study was conducted with the approval of the University of Oregon Institutional Review Board. Written assent was obtained from all participants under 18 years of age, and their parents signed a consent form for their children. Participants older than 18 years of age signed a consent form to participate. All participants were paid for their time.

#### Stimuli and Procedure

The task was a modification of the change detection paradigm used in Luck and Vogel (1997). Stimuli consisted of colored squares (0.65° × 0.65°) superimposed on stick figures, introduced to the participants as children wearing colored shirts. Each colored square was selected at random from a set of nine colors (red, pink, violet, blue, green, yellow, orange, brown, and black). A given color did not appear more than once within an array. The memory arrays included set sizes of 2, 4, or 6 stimuli. The items in a given array were separated by at least 3° from the center of each square to the center of the other. The positions of the stimuli were randomized on each trial to appear within a 9.8° × 7.3° region on a monitor with a gray background, at a viewing distance of 75 cm. A fixation cross was presented at the center of the screen throughout the study.

**FIGURE 1:**
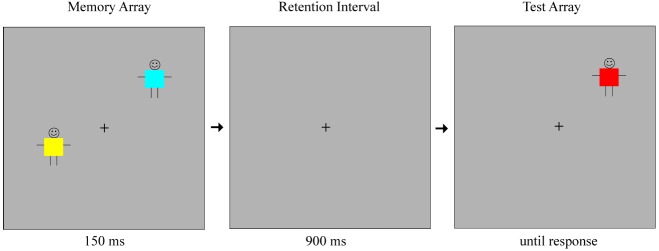
**Example stimulus displays (not drawn to scale) for a “change trial” of set size 2**.

On each trial, the first array of stimuli (the memory array) was presented for 150 ms, followed by a 900 ms blank retention interval. Example stimuli are shown in Figure 1. After the retention interval, only one object reappeared on the screen. In half of the trials, this object was identical to the object that appeared in the same location within the memory array. In the other half of the trials, the object was a different color from the object that appeared in the same location before. This was always a new color, not presented elsewhere in the display within the memory array. Participants were informed that in each trial a group of children wearing different colored shirts were going to come up on the screen, disappear briefly, and then only one child would come back to the screen, in the exact location he was before. They were asked to indicate whether the child was wearing the same shirt or had changed his shirt. The participants indicated their responses using the left and right triggers of a video game controller, which were marked as “same” and “change” respectively. The test item remained on the screen until a response was made.

A practice block was administered before the main task to demonstrate the task to participants and allow them to get more comfortable using the interface. The practice block consisted of six trials of set size 2, followed by six trials of set size 4. If a participant performed below 66% accuracy for either set size, the practice block was repeated. No participant had to repeat the practice block more than twice. The experiment consisted of 80 trials of each set size presented randomly, for a total of 240 trials. Participants were offered a break every 80 trials. The experiment took approximately 12 min to complete.

#### Data Analysis

Visual working memory capacity was calculated as *K* = *S(H – F)*, where *K* is the VWM capacity, *S* is the set size of the visual array, *H* is the hit rate, and *F* is the false alarm rate (Cowan, 2001). Univariate ANOVAs were used to examine the omnibus effects of age. Planned contrasts were employed to compare the 13-year-olds versus adults, and 16-year-olds versus adults. For all planned contrasts with *p* < 0.05, Cohen’s *d* (Cohen, 1977; Rosnow et al., 2000) was reported as the measure of effect size.

### Results and Discussion

Capacity estimates obtained from the set size 2 condition resulted in a potential underestimation (i.e., *K* < 2.00) of VWM capacity (Rouder et al., 2008). Nevertheless, to demonstrate that our results did not depend on the exclusion of this condition, we first conducted all analyses including the set size 2 condition in the grand averages of *K*. Means and standard deviations of *K* estimates are reported in Table 1.

**TABLE 1:** **Descriptives of VWM capacity (*K*) estimates for the three age groups in Experiment 1**.

****	**13-year-olds (*n* = 22)**		**16-year-olds (*n* = 22)**		**Adults (*n* = 23)**	
**Set size condition**	***M***	**(SD)**	***M***	**(SD)**	***M***	**(SD)**
SS2	1.74	(0.18)	1.83	(0.15)	1.89	(0.10)
SS4	2.25	(0.77)	2.64	(0.57)	2.93	(0.52)
SS6	2.18	(0.92)	2.80	(0.77)	3.38	(0.71)

**FIGURE 2:**
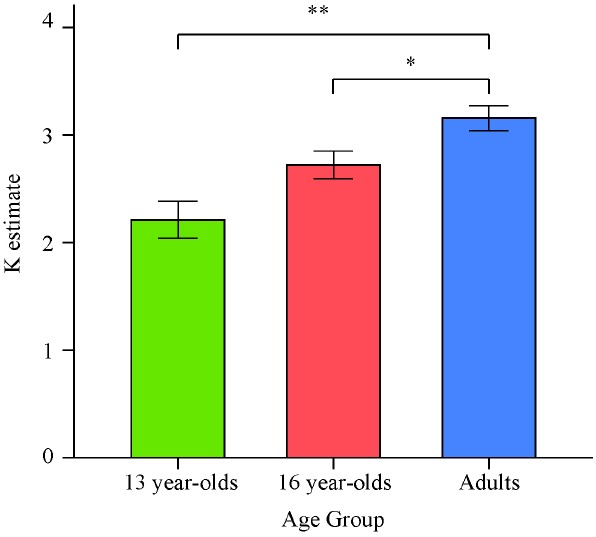
**Means and standard errors of the average VWM capacity (*K*) estimates computed based on set sizes 4 and 6 for the three age groups in Experiment 1.** *p < 0.05; **p < 0.001.

There was a significant effect of age on *K* estimates obtained as an average from all set sizes, *F*(2,64) = 11.68, *p* < 0.001, ${\text{η}}_{p}^{2}$ = 0.27. Planned contrasts revealed that the 13-year-olds had lower estimates than adults, *t*(64) = –4.86, *p* < 0.001, *d* = –1.42. Critically, 16-year-olds were also found to perform worse than adults, *t*(64) = –2.20, *p* = 0.031, *d* = –0.78. However, since the set size 2 condition lowered the *K* estimates for each group and the direction of the results did not appear to depend on the inclusion of this condition, we excluded this condition in a second analysis of the effect of age on overall *K*. The *K* estimates obtained as an average of the set size 4 and set size 6 conditions for the three age groups are illustrated in Figure 2.

For the *K* estimates excluding the set size 2 condition, there was again a significant effect of age on performance, *F*(2,64) = 11.41, *p* < 0.001, ${\text{η}}_{p}^{2}$ = 0.26. Consistent with the previous findings, planned contrasts revealed that both 13- and 16-year-olds had lower VWM capacity estimates than adults [*t*(64) = –4.77, *p* < 0.001, *d* = –1.40, and *t*(64) = –2.19, *p* = 0.032, *d* = –0.76, respectively].

Replicating previous findings with young adolescents (Cowan et al., 2006; Spronk et al., 2012), this experiment demonstrated that 13-year-olds do not have adult-like VWM capacity estimates. Expanding on these findings, this experiment also showed that even 16-year-olds have lower capacity estimates than adults. These results suggest that VWM capacity does not reach adult levels in adolescence.

## Experiment 2

### Introduction

Experiment 1 provided confirmatory evidence that the estimates of VWM capacity do not reach adult levels in early adolescence, and the first evidence that they may not reach adult levels even in late adolescence. These results contradict the claims that VWM capacity reaches adult-levels during early years of life. However, it is possible that the discrepancy of findings between these studies is at least partially driven by differences in the duration of memory arrays. In the studies that claimed the VWM capacity reached adult levels in early years of life (Ross-Sheehy et al., 2003; Riggs et al., 2006), the memory arrays were presented for 500 ms. However, we presented the memory arrays for 150 ms in Experiment 1. To rule out the possibility that the poorer performance of the adolescents was mainly driven by a lack of sufficient exposure to the memory array, in Experiment 2, we presented the memory arrays for both short and longer durations.

There are contradictory findings on the effects of increased presentation time on VWM performance. In a study with young adults, increasing the duration of the memory array from 100 to 500 ms was not found to improve VWM performance (Vogel et al., 2001). On the contrary, in a study that compared the VWM performance in children, younger adults, and older adults, performance was found to increase from 100 to 500 ms (but not from 500 to 1000 ms) for all age groups (Sander et al., 2011).

In this experiment, we explored the effects of the duration of the memory array on VWM in adolescents and adults. We aimed to replicate the findings of Experiment 1 and also determine (a) whether the adolescents benefited more than adults from longer exposure times; and (b) whether the increase in exposure to the memory array was sufficient to eliminate the age differences in performance observed in the first experiment. In order to examine the effects of exposure time on the VWM performance of adolescents and adults, we presented the participants with memory array durations of 150, 500, and 1000 ms, randomly presented across trials. We did not use a presentation time longer than 1000 ms to prevent the use of verbal encoding during memory arrays.

An additional strength of Experiment 2, relative to Experiment 1, is the use of a more powerful statistical technique, multilevel modeling (MLM), to analyze the data. Multilevel modeling is appropriate in this case because our data are structured as responses within individuals, with duration as a within-person independent variable and age as a between-person independent variable. Typically the clustering of responses with individuals in repeated-measures design is addressed by averaging the responses but this approach discards potentially meaningful variability at the within-person level. Here, MLM allows us to analyze all responses for all durations and all participants in a single, powerful model.

### Method

#### Participants

The final sample included twenty-nine 13-year-olds (*M* = 13.41 years, SD = 0.25; 14 females), twenty-eight 16-year-olds (*M* = 16.48 years, SD = 0.29; 15 females), and 32 adults (*M* = 20.58 years, SD = 2.09; 15 females). All participants had normal or corrected-to-normal vision and were typically developing individuals with no neurological disorders, ADD/ADHD, learning disabilities, color blindness, or visual tracking problems. All adolescent participants were middle school and high school students, attending a variety of schools in Eugene, Oregon. All adult participants were University of Oregon undergraduates. One 13-year-old participant and one 16-year-old participant were not included in this final sample for performing below chance at higher set sizes, suggesting that these participants were not fully engaged in the task.

Maternal education levels were compared as a proxy for SES and no differences were observed between groups [*F*(2,78) = 0.52, *p* = 0.60]. The average maternal education level corresponded to “completed some college classes” across age groups.

The study was conducted with the approval of the University of Oregon Institutional Review Board. Written assent was obtained from all participants under 18 years of age, and their parents signed a consent form for their children. Participants older than 18 years of age signed a consent form to participate. All participants were paid for their time.

#### Stimuli and Procedure

The paradigm described in Experiment 1 was modified to investigate the effects of memory array duration on performance. As in the first experiment, the memory arrays consisted of 2, 4, or 6 items on display. In Experiment 1, *K* estimates from the set size 2 condition was found to lower the overall capacity estimates but the direction of the results did not appear to depend on the inclusion of this condition. We kept the set size 2 condition in the experiment to parallel the design from Experiment 1 as closely as possible. The memory arrays were presented for 150, 500, or 1000 ms. The experiment consisted of 120 trials of each presentation time, with both the set sizes and memory array durations randomized across trials. There were a total of 360 trials. Participants were offered a break every 90 trials, with a potential of taking three breaks during the study. The experiment took approximately 20 min to complete.

#### Data Analysis

Response time data are clustered within subjects in the sense that observations from the same participant are more highly correlated with each other than observations from different participants. This violates the general linear model assumption of independence of errors at the response level. MLM explicitly addresses this issue by separately estimating the within- and between-subject error under the assumption that within-subject observations are not independent (Garson, 2013). Accordingly, we used MLM with VWM capacity estimates nested in individuals for this repeated measures design. The multilevel model was analyzed with Hierarchical Linear Modeling (HLM) software (Raudenbush and Bryk, 2002). The within-person predictor, which was duration, was entered at Level 1, and the between-person predictor, age group, was entered at Level 2. We used an unstructured variance/covariance matrix to allow for heterogeneous errors across age groups. Exposure time was centered at 150 ms, and dummy codes were used to compare 13-year-olds to adults as well as 16-year-olds to adults. The following model was used where the intercept and the slopes were allowed to vary randomly.

$$\begin{array}{ll} \text{Level 1:} & Reponse_{ij}=\beta_{0i}+\beta_{1i}\;TIME_{ij}+{\text{e}}_{ij}\\ \text{Level 2:} & \beta_{0i}=\gamma_{00}+\gamma_{01}*\left(Early\;Adolescence\right)+\gamma_{02}*\left(Late\;Adolescence\right)+u_{0i}\\ & \beta_{1i}=\gamma_{10}+\gamma_{11}*\left(Early\;Adolescence\right)+\gamma_{12}*\left(Late\;Adolescence\right)+u_{1i} \end{array}$$

In this model, *Response_ij_* is predicted by a linear function of exposure time (*TIME_ij_*) for the VWM capacity estimate of individual *i* at occasion *j*. The intercept (*β_0i_*) represents the individual *i*’s VWM capacity estimate at 150 ms. The slope (*β_1i_*) represents the effect of exposure time on an individual’s VWM capacity estimate. This multilevel model allowed us to test for replication of the findings from Experiment 1 at 150 ms (the parameters in the *β*_0_ equation), and additionally, whether there was a main effect of exposure time (*γ*_10_) and if the age groups differentially benefited from longer exposure to the memory arrays (*γ*_11_ and *γ*_12_). Cohen’s *d* (Cohen, 1977; Rosnow et al., 2000) is reported for the comparison of the dummy-coded groups at the intercept (150 ms).

### Results and Discussion

**FIGURE 3:**
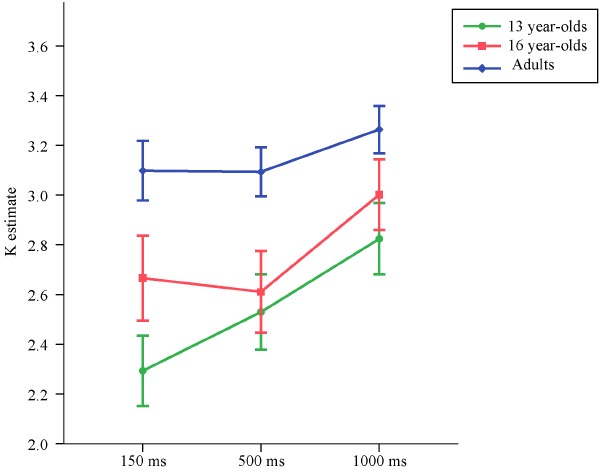
**Means and standard errors of the VWM capacity (*K*) estimates computed based on set sizes 4 and 6 for the three age groups across exposure conditions in Experiment 2**.

Means and standard deviations of *K* estimates are reported in Table 2, separately for set size 4, set size 6, and the average of the *K* estimates from set size 4 and set size 6. The average *K* estimates for the three age groups across exposure conditions are illustrated in Figure 3.

**TABLE 2:** **Descriptives of VWM capacity (*K*) estimates across exposure conditions in Experiment 2**.

****	**13-year-olds (*n* = 29)**	**16-year-olds (*n* = 28)**	**Adults (*n* = 32)**
**Exposure condition**	***M* (SD)**	***M* (SD)**	***M* (SD)**
**150 ms**			
SS4	2.33 (0.68)	2.64 (0.78)	3.00 (0.57)
SS6	2.26 (1.14)	2.69 (1.23)	3.20 (0.98)
Average *K*	2.29 (0.76)	2.67 (0.90)	3.10 (0.68)
**500 ms**			
SS4	2.59 (0.64)	2.65 (0.69)	3.02 (0.55)
SS6	2.47 (1.14)	2.57 (1.22)	3.17 (0.78)
Average *K*	2.53 (0.82)	2.61 (0.87)	3.10 (0.57)
**1000 ms**			
SS4	2.81 (0.64)	2.84 (0.67)	3.16 (0.53)
SS6	2.83 (1.06)	3.16 (1.10)	3.37 (0.83)
Average *K*	2.82 (0.77)	3.00 (0.75)	3.26 (0.54)

Although the capacity estimates obtained from the set size 2 condition results in underestimation of VWM capacity, to demonstrate that our results did not depend on the exclusion of this condition, once again we first conducted all analyses including the set size 2 condition in the grand averages of *K*.

Replicating the results from Experiment 1, at 150 ms exposure time the 13-year-olds performed worse than adults, *t*(86) = –3.92, *p* < 0.001, *d* = –1.15. Similarly, in this shortest duration condition, 16-year-olds also performed worse than adults, *t*(86) = –2.31, *p* = 0.023, *d* = –0.57. Duration of the memory array did not have a significant effect on the increase in VWM capacity in adults, *t*(86) = 1.54, *p* = 0.12. There was a significant difference in the rate of increase in VWM capacity between 13-year-olds and adults, *t*(86) = 2.31, *p* = 0.023. The rate of increase in capacity over exposure time did not differ between 16-year-olds and adults, *t*(86) = 1.13, *p* = 0.26.

Similarly, for the *K* estimates excluding the set size 2 condition, at 150 ms exposure time adults had higher capacity estimates than both the 13-year-olds [*t*(86) = 3.86, *p* < 0.001, *d* = 1.14] and the 16-year-olds [*t*(86) = 2.50, *p* = 0.015, *d* = 0.56].

Also for the *K* estimates excluding the set size 2 condition, duration of the memory array did not have a significant effect on the increase in VWM capacity in adults, *t*(86) = 1.71, *p* = 0.090. Unlike the analyses that included set size 2 condition, the difference in the rate of increase in VWM capacity between 13-year-olds and adults did not reach significance at the *p* < 0.05 level, but was very close to this alpha cut-off, *t*(86) = 1.97, *p* = 0.051. The rate of increase in capacity over presentation time did not differ between 16-year-olds and adults, *t*(86) = 1.31, *p* = 0.19.

To test whether 16-year-olds performed worse than adults even at the longest presentation condition, for the *K* estimates excluding the set size 2 condition which results in underestimation of capacity, a subsequent model was run, once again with heterogeneous error terms. To have a parsimonious model, as the rate of increase in VWM over presentation time did not differ significantly between the 16-year-olds and adults, age group was included only as a predictor of the capacity estimates at 1000 ms, but not as a predictor of the rate of decrease in capacity. This model revealed that even at 1000 ms, 16-year-olds performed worse than adults, *t*(58) = –2.20, *p* = 0.032, *d* = –0.41.

While Vogel et al. (2001) reported no improvement in capacity from 100 to 500 ms in young adults, Sander et al. (2011) found an increase in capacity for 10-year-olds, young adults, and older adults from 100 to 500 ms, but not from 500 to 1000 ms. In this experiment, we did not find a significant effect of exposure time for young adults. However, an interesting pattern for the slope of increase in capacity was observed when the younger and older adolescents were compared to the adults. As shown in Figure 3, 13-year-olds showed the greatest rate of improvement in capacity with longer exposure to the memory array. Younger adolescents appeared to benefit differentially from longer exposure time than older participants, who did not seem to benefit much from an increase in the duration of the memory array. These findings imply that different factors account for why younger adolescents do not perform at adult levels as compared to older adolescents. It is possible that an immature profile in identifying and transferring perceptual representations into VWM partially accounts for the poorer performance of younger adolescents, while older adolescents do not display such an immaturity in encoding processes. In addition, the maturation levels of cortical structures that show a temporal activation profile not accounted for by perceptual or general attention effects, such as the inferior frontal junction in the lateral prefrontal cortex (Todd et al., 2011), may account for why younger adolescents differentially benefit from longer exposure to the memory arrays.

Despite differentially benefiting from longer exposure to the memory array, both younger and older adolescents still performed worse than young adults. Longer exposure to the memory array diminished but did not eliminate the age differences in VWM capacity observed in Experiment 1. These findings suggest that the capacity differences observed between adolescents and adults in Experiment 1 were not driven solely by short presentation times and support the hypothesis that VWM capacity has a prolonged developmental trajectory.

## General Discussion

The present study investigated whether VWM capacity continues to develop through adolescence into adulthood. Overall, our results demonstrated that the capacity of VWM does not reach adult levels either in earlier or later years of adolescence. Regardless of whether the memory array was presented briefly or for longer durations, neither younger nor older adolescents displayed adult-like capacity estimates. Our findings are consistent with previous studies that demonstrated lower capacity estimates in early years of adolescence compared to adulthood (Cowan et al., 2005; Spronk et al., 2012). Here we extend these findings to later years of adolescence, in line with the claim that VWM performance improves throughout adolescence (Brockmole and Logie, 2013).

Our results contradict the assertions that visual WM capacity reaches adult levels in infancy (Ross-Sheehy et al., 2003; Oakes and Luck, 2014) or middle childhood (Riggs et al., 2006). It is possible that VWM capacity does not develop in a linear fashion, but rather follows a U-shaped developmental trajectory, reaching higher levels of performance earlier in life followed by a dip in performance during adolescence, and resurgence into adulthood. Indeed, there are examples of such non-linear developmental trajectories in other aspects of cognition (Uhlhaas et al., 2009; Dumontheil et al., 2010).

However, it is also likely that the discrepancy of findings between these infant studies and the other developmental studies of VWM capacity stems from paradigm differences. In studies that employed a variation of the change detection task (Luck and Vogel, 1997) with children, adolescents, and adults, participants have been asked to verbally or manually respond to indicate whether a change occurred in the display (Cowan et al., 2005, 2006; Sander et al., 2011; Spronk et al., 2012). However, in infant studies, VWM capacity has been assessed predominantly with gaze behavior of infants (Ross-Sheehy et al., 2003; Oakes et al., 2013; Kwon et al., 2014). We cannot rule out the possibility that the response characteristics of paradigms play a role in VWM capacity estimates obtained in each study. It has been argued that looking time paradigms may tap into different cognitive processes compared to tasks with overt response demands, yielding differential performance profiles (Karmiloff-Smith, 1992; Hood et al., 2000; Keen, 2003; Lee and Kuhlmeier, 2013). It is plausible that variations of VWM paradigms, regardless of how similar they appear, may have inherent differences in what aspects of VWM they measure. In fact, studies that tested children, adolescents, and adults with similar tasks and similar methods of response acquisition consistently demonstrated lower VWM capacity estimates in children and young adolescents as compared to adults (Cowan et al., 2005, 2006; Sander et al., 2011; Spronk et al., 2012). Our results expand the findings of these studies and suggest that VWM performance develops through later years of adolescence into adulthood.

Several studies reported developmental changes for verbal and spatial working memory span tasks throughout adolescence (Kwon et al., 2002; Gathercole et al., 2004; Luna et al., 2004; Luciana et al., 2005; Johnson et al., 2014). Furthermore, developmental changes in adolescence were observed for various other aspects of cognition, such as decision-making (Crone and van der Molen, 2004), speed of processing (Kail, 1991; Ferrer et al., 2013), creative thinking (Kleibeuker et al., 2013), and reasoning (Huizenga et al., 2007; Ferrer et al., 2013). Our results suggest that the VWM capacity shows a prolonged development in adolescence, similar to the trajectories observed in other aspects of working memory, as well as various other cognitive abilities.

Although our study provides evidence for age related differences in VWM capacity between adolescents and adults, the mechanisms underlying such differences require further investigation. Adolescence is a pivotal period for brain development during which substantial changes are observed (Lebel and Beaulieu, 2011; Raznahan et al., 2011; Blakemore, 2012; Klein et al., 2014). Previous research associated changes in brain functioning from adolescence to adulthood with developmental changes in visuospatial working memory performance (Kwon et al., 2002; Scherf et al., 2006; Bunge and Wright, 2007). It is plausible that changes in VWM capacity estimates from adolescence to adulthood are driven by functional alterations in the cortical regions that are involved in working memory processes such as the parietal cortex and prefrontal cortex (Curtis and D’Esposito, 2003; Todd and Marois, 2004). In addition, the size and density of white matter tracts connecting prefrontal, occipital, parietal, and temporal lobes have been linked to VWM capacity (Golestani et al., 2014). As white matter microstructures drastically transform throughout adolescence (Nagy et al., 2004; Barnea-Goraly et al., 2005; Mabbott et al., 2006), alterations in white matter from adolescence through adulthood may also account for developmental changes in VWM capacity.

Potentially, the age related differences in VWM capacity estimates may stem from disparities in attention skills rather than genuine differences in the number of slots available in VWM. Attentional control has been postulated as a critical component of working memory (Engle and Kane, 2004). In support of this view, poorer attentional control has been linked to lower VWM capacity estimates (Vogel et al., 2005; Fukuda and Vogel, 2009, 2011; Unsworth and Robison, 2014). In this regard, having lower capacity estimates on average, adolescents may actually resemble low capacity adults. Research with adults demonstrated that low-capacity adults have poorer filtering skills, which prevents them from excluding irrelevant items from VWM (Vogel et al., 2005). Furthermore, low-capacity adults are found to recover from attentional capture more slowly than high-capacity adults (Fukuda and Vogel, 2011). If adolescents are more like low capacity adults in performance, the poorer performance they exhibit may be a result of their inefficiency in using the available slots for VWM. In line with this claim, in an event-related potentials (ERP) study with adolescents and adults, contralateral delay activity (CDA) was found to be larger in adolescents than adults when there were one target and two distractor items, as opposed to the similar CDA observed when there was only a target item on display (Spronk et al., 2012).

However, the mechanisms responsible for the poorer performance of adolescents and low capacity adults may also be distinct from each other. For instance, in a study comparing older adults to younger adults, older adults were not simply like low capacity young adults, despite performing worse than younger adults on average (Jost et al., 2011). Similarly, in spite of the similarities in capacity estimates between adolescents and lower capacity adults, there may be differential mechanisms driving such poor performance.

Alternatively, the observed differences in performance may stem from age related disparities in the number of slots available in VWM. A recent study with children investigated whether such disparities in available slots account for differences in performance between children and adults (Cowan et al., 2010). It was argued that inefficiency of attention cannot fully explain the observed age differences in performance and that there were genuine storage differences between children and adults. Accordingly, there may be differences in how many slots are available in VWM for adolescents as compared to adults. Moreover, there may be different underlying mechanisms that result in immature profiles of VWM in younger and older adolescents. Our results suggested that younger adolescents benefited more from longer exposure to memory displays than adults, while older adolescents did not show such benefits. These results imply different limiting factors for performance in earlier and later years of adolescence.

In addition to differing in VWM capacity estimates, adolescents may differ from adults in the resolution of VWM representations. It has been demonstrated that the number of items held in mind for immediate access and the resolution of these representations are distinct aspects of VWM (Xu and Chun, 2006; Awh et al., 2007; Fukuda et al., 2010). Therefore, there may be distinct developmental trajectories for how many items can be held in working memory versus how precise these representations are. While the number of items held in memory increase with age, the precision of these representations may reach adult levels earlier during development. On the contrary, regardless of different underlying neural mechanisms (Xu and Chun, 2006), both systems that support VWM may appear immature in adolescence.

It is important to note that in both studies we compared adolescents to young adults, who were on average 20 years of age. Although it has been argued that VWM performance peaks at age 20 (Brockmole and Logie, 2013), we cannot ascertain that the young adults in our study reflect the peak VWM performance in adulthood. It is possible that VWM continues to develop into the third decade of life, reflecting structural changes in brain maturation in adulthood (Sowell et al., 2001, 2003; Lebel and Beaulieu, 2011). In addition, all of the adult participants in our experiments were college students. We matched the adolescents and adults in our study based on maternal education levels. However, it should be noted that the maternal education levels in our samples were relatively high, corresponding to at least some post-secondary education. Therefore, it remains to be assessed how our results would generalize to both youth and adults from diverse SES backgrounds. Moreover, a more comprehensive battery of cognitive measures would be required to rule out any confounding cognitive differences between adolescents and adults. Future studies that include a wider range of age and SES and more detailed cognitive assessments can greatly benefit the investigation of typical VWM development from adolescence into adulthood. Furthermore, incorporating neuroimaging methods can assist in determining the factors that account for age related differences in VWM capacity estimates.

Although much remains to be investigated, our study provides evidence for a protracted developmental profile of VWM capacity. As a late developing system that does not appear to reach adult levels even in late adolescence, VWM capacity bears the potential to be a rather plastic system in development, malleable to the effects of the environment. Studies on neuroplasticity across development have repeatedly demonstrated that plastic systems can both be compromised and enhanced depending on experience (Stevens and Neville, 2009). Therefore, deficiencies in VWM may be found in adolescents who have experienced adversity through development. For instance, lower maternal education has been associated with poorer WM performance in adolescents and these associations appear to be stable through adolescence (Hackman et al., 2014). Targeted screenings and interventions to follow may be helpful in mitigating such disparities. Drawing parallels from studies that show children with poor WM skills especially benefit from adaptive WM training (Holmes et al., 2009), targeted trainings may be particularly effective for adolescents with lower VWM capacity. Since VWM capacity is a predictor of academic achievement in children, interventions that aim to improve VWM skills may eventually become helpful tools in improving the academic outcomes of adolescents who are at risk for school failure.

### Conflict of Interest Statement

The authors declare that the research was conducted in the absence of any commercial or financial relationships that could be construed as a potential conflict of interest.
